# Myomesin is part of an integrity pathway that responds to sarcomere damage and disease

**DOI:** 10.1371/journal.pone.0224206

**Published:** 2019-10-23

**Authors:** Kendal Prill, Casey Carlisle, Megan Stannard, Pamela J. Windsor Reid, David B. Pilgrim

**Affiliations:** 1 Department of Molecular and Cellular Biology, University of Guelph, Guelph, Ontario, Canada; 2 Department of Molecular Genetics, University of Toronto, Toronto, Ontario, Canada; 3 Department of Biological Sciences, University of Alberta, Edmonton, Alberta, Canada; 4 Department of Biological Sciences, MacEwan University, Edmonton, Alberta, Canada; University of Maryland Center for Environmental Science, UNITED STATES

## Abstract

The structure and function of the sarcomere of striated muscle is well studied but the steps of sarcomere assembly and maintenance remain under-characterized. With the aid of chaperones and factors of the protein quality control system, muscle proteins can be folded and assembled into the contractile apparatus of the sarcomere. When sarcomere assembly is incomplete or the sarcomere becomes damaged, suites of chaperones and maintenance factors respond to repair the sarcomere. Here we show evidence of the importance of the M-line proteins, specifically myomesin, in the monitoring of sarcomere assembly and integrity in previously characterized zebrafish muscle mutants. We show that myomesin is one of the last proteins to be incorporated into the assembling sarcomere, and that in skeletal muscle, its incorporation requires connections with both titin and myosin. In diseased zebrafish sarcomeres, *myomesin1a* shows an early increase of gene expression, hours before chaperones respond to damaged muscle. We found that myomesin expression is also more specific to sarcomere damage than muscle creatine kinase, and our results and others support the use of myomesin assays as an early, specific, method of detecting muscle damage.

## Introduction

Muscle tissue is composed of bundles of myofibrils that are assembled from tandem repeats of the contractile unit, the sarcomere [1–3]; a complex structure of contracting and relaxing sliding filaments. Hundreds of components are involved in building the sarcomere [1–5], which can be roughly grouped into contractile and structural proteins [2–4,6–14], and proteins involved in the assembly and maintenance of the sarcomere (such as chaperones) but not necessarily present in the assembled complex [11,15–23]. The architecture, composition and function of the mature sarcomere is largely well characterized but the steps involved in assembling this complex structure are still largely unresolved. In particular, the structure of the part of the sarcomere known as the M-line remains poorly understood. The M-line anchors parts of the contractile sarcomere (myosin and titn) and contains proteins such as myomesin and obscurin. Titin is the most extensively studied of the M-line proteins and mutations in titin’s C-terminus lead to limb girdle muscular dystrophies, tibial and Salih congenital muscular dystrophies [9,24,25]. Although a mutation in the dimerization domain of myomesin 1 leads to hypertrophic cardiomyopathy in humans, no other mutations have been described in M-line proteins, including myomesin 2 and 3 [26]. The zebrafish experimental system allows us to address these questions *in vivo* as fish with ultimately fatal myopathies can still survive to the end of embryogenesis, as heart function is not required for these stages of development. Another advantage is that transparent embryos develop externally allowing us to visualize the defective sarcomere as the organism grows [27–31].

**Fig 1:**
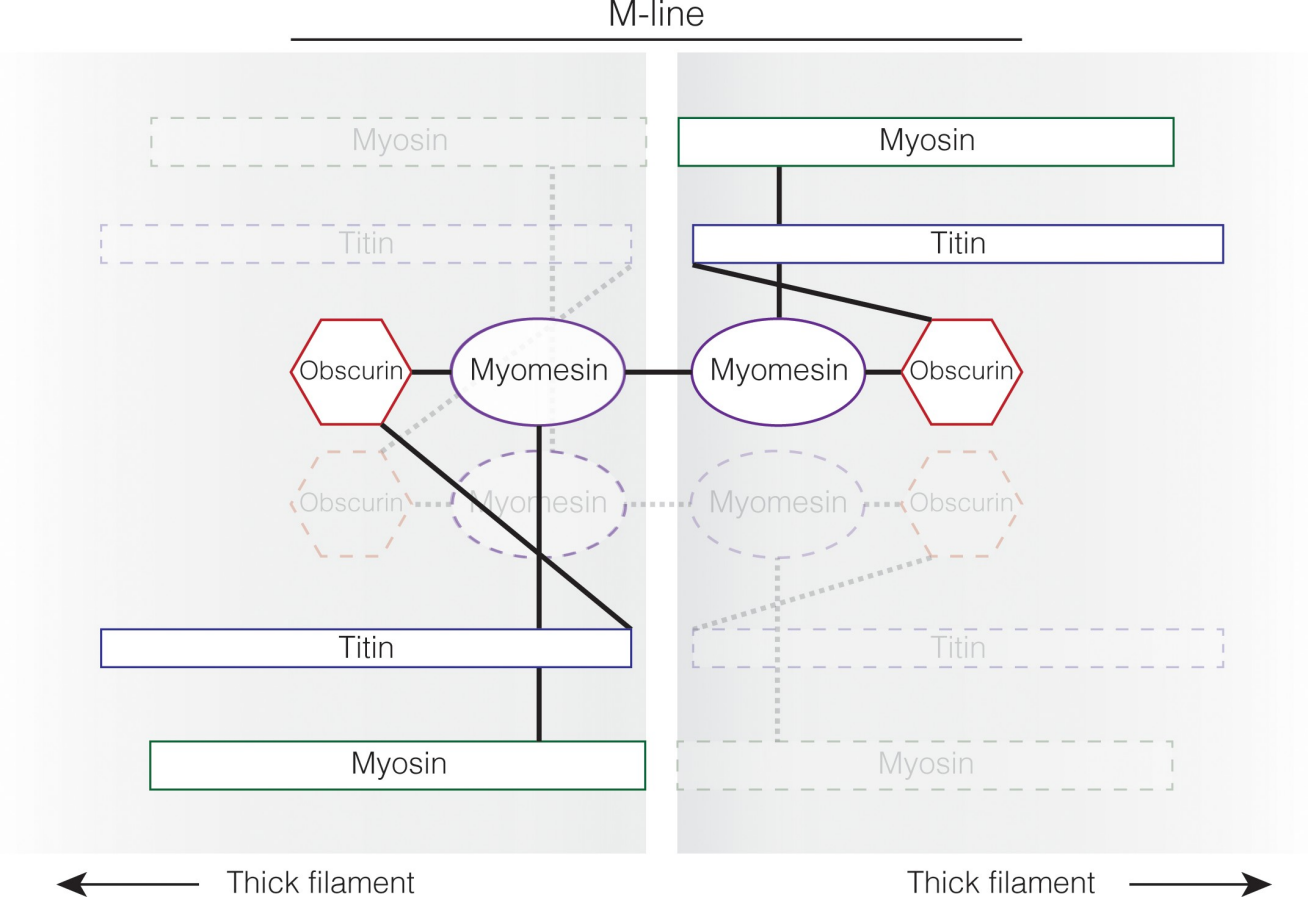
The sarcomere proteins of the M-line and their physical interactions. The M-line of the sarcomere diagraming the protein interactions between myosin thick filaments. The physical protein interactions through the M-line between antiparallel thick filaments are demonstrated by solid lines for one connection and faded dotted lines for the other connection between antiparallel thick filaments. Myosin and titin are incorporated into the M-line around the same time. This work suggests that myomesin is added next and requires both titin and myosin to be present for myomesin to be incorporated. Titin and myomesin together recruit obscurin, or obscurin-like 1, to the M-line [9,42].

In addition to binding terminal domains of titin and myosin, the M-line is composed of three major structural proteins, obscurin, obscurin-like 1, and myomesin (Fig 1); although each protein may have different isoforms in different muscle types and/or at different developmental stages [6,32–34]. Titin, myomesin and myosin form a network at the center of the sarcomere to anchor thick filaments within the A-band. Myomesin, obscurin and obscurin-like 1 connect thick filament bundles throughout the sarcomere and equalize the contractile force exerted by the thick filaments during contraction [6,35]. Myosin must make attachments with the M-line to be correctly positioned within the sarcomere for contraction. Titin must also attach to the M-line to provide an elastic “spring” between the Z-disc and M-line [7,8,36–38]. Additionally, it seems that myosin and titin connections are needed to also hold the M-line together [9,37]. The N-terminus of myomesin attaches to the tails of the myosin thick filaments while the C-terminus allows for dimerization of myomesin proteins within the M-line [39]. The C-terminus of titin makes contact with myomesin and loosely holds several myomesin proteins together [17,36,37]. All of these contacts are critical to the M-line and disruption of one leads to degeneration of the sarcomere [9,17,36]. If one component of the M-line is not present, or all of the above connections cannot be made, the A-band ultimately collapses leading to muscle paralysis [11,20,22,23,40,41].

Myomesin is not required for myosin to be targeted or incorporated into the sarcomere [43], but myosin is required for myomesin incorporation to the M-line, therefore defects in myosin assembly into the sarcomere (such as due to lack of myosin chaperones) may indirectly abolish myomesin incorporation [43,44]. Titin is also required to maintain myomesin in the M-line as observed in animals with C-terminal titin mutations, myomesin is absent from the sarcomere. Recently, myomesin 3 has been proposed to be a more sensitive serum biomarker of muscle damage than creatine kinase [45]. We hypothesize that myomesin incorporation into the sarcomere is essential to maintain muscle health and that the M-band is sensitive to any disruptions in this process. If this hypothesis is correct, we expect myomesin would be one of the last sarcomere proteins to incorporate as an “integrity check” that sarcomere formation is normal. We would also expect that myomesin mRNA expression would increase in response to either auto-regulation or transcriptional activation as part of a repair pathway due to the absence of an M-line structural protein.

Here we show that myomesin is one of the last proteins to appear in the developing sarcomere, and myomesin fails to incorporate in the skeletal sarcomeres of the zebrafish myosin chaperone mutants *still heart* and *steif*, and the zebrafish titin mutant, *herzschlag*. We also observed a strong increase in *myomesin1a (myom1a)* expression at the stage of thick filament assembly in these mutants. This dramatic increase in *myom1a* expression signifies a myomesin-dependent response pathway to sarcomere damage at the earliest stages of muscle disease, as this expression is detectable earlier than myosin chaperone expression. These results support the use of myomesin as an early detection method of sarcomere damage in striated muscle disease.

## Materials and methods

### Ethics statement

All procedures/methods were performed following the guidelines stated by the Canadian Council for Animal Care and the protocols approved by the Animal Care and Use Committee of the University of Alberta (Fish Research License: 14–0101 FR).

### Zebrafish husbandry and strain maintenance

All adult zebrafish were bred and maintained according to standard procedures [46], and kept at 28°C on a day/night cycle of 14 hours light/10 hours dark. Adults were housed in a cycled-water aquatic facility and fed twice daily with brine shrimp. Embryos were raised at 28°C in standard embryo medium [46] for up to 3 days prior to fixation.

The mutants *still heart* (referred to as *smyd1b*^*tm123a*^) and *herzschlag* (referred to as *titin2*^*tg287*^) were initially identified by the Nusslein-Volhard lab from an ENU mutagenesis screen and further characterized by our lab [22,40,47,48]. The *unc45b*^*sb60*^ mutants (*steif*), which were also generated from a ENU mutagenesis screen, were a generous gift from Dr. Uwe Strahle [19]. All mutant lines were maintained as adult heterozygotes in a recycling aquatics facility [46]. Embryos were collected from the crossing of two adult heterozygotes and maintained at 28.5°C in zebrafish embryo media before collection for experimental procedures.

### *In situ* hybridization and immunostaining

*In situ* hybridization were performed using whole-mount zebrafish embryos fixed overnight in 4% paraformaldehyde at 4°C. Antisense RNA probes were synthesized by *in vitro* transcription using T7 RNA polymerase. Probes used were synthesized from the 3’ untranslated region of *myom1a* (GenBank:AL929096; FWD- CACAGAGAGCCAATACAAC and REV- TCCTCTACATCAGCATCTC) and *ckmb* (GenBank:CR855332; FWD- GTGCCATCATGACTTTTCC and REV- GGTGAATACAACACAGCTAGG). For immunostaining, embryos were staged morphologically and fixed in 4% paraformaldehyde overnight at 4°C. The following day, embryos were transferred into PBST and allowed to permeabilize for 2 days at 4°C before beginning antibody staining. Embryos were washed 3 x 15 mins in PBST before permeabilization in ice-cold acetone at -20°C (30 mins– 24hpf; 45 mins– 32hpf; 1 hour– 48hpf; no acetone for heart immunostaining). Embryos were washed 4 x 15 mins in PBST and then incubated in 5% goat serum+PBST for 1 hour at room temperature. After goat serum incubation, embryos were incubated with primary antibody, myomesin (mMacB4, AB_760349, DSHB), slow myosin (F59, AB_528373, DSHB) or titin (T11, AB_2211848, Thermofisher Scientific) in 5% goat serum+PBST overnight at 4°C. The following day, primary antibody solution was removed and embryos were washed 4 x 15 mins in PBST. Following PBST washes, 5% secondary antibody (Alexa Fluor 488 goat anti-mouse, AB_2534069, Thermofisher Scientific) +1% phalloidin (Alexa Fluor 568, Thermofisher Scientific) in PBST was added to the embryos and incubated overnight at 4°C. The following day, secondary was removed and the embryos were washed 3 x 10 mins at room temperature. Immunofluorescence was imaged on a Nikon C2 Confocal Microscope.

### RNA extractions and qPCR

RNA extractions and cDNA preparation were carried out as previously described [22] (*myom1a* qPCR primers: FWD- AGGTTGCTATAGCCAATGTGAT and REV- GCATTAAGCAAGATATATCAGCAGAC).

### Tricaine drug treatment

The paralysis of wild-type, *smyd1b*^*tm123a*^, *unc45b*^*sb60*^ and *titin2*^*tg287*^ mutants was carried out using Tricaine or MS-222 (Sigma Aldrich). Stock solutions of Tricaine were prepared by adding 400 mg tricaine to 98 ml of distilled water in low light. Stock solutions were vortexed until mixed thoroughly and then frozen until needed. Working solutions were prepared by adding 4.2 ml of Tricaine stock solution to 50 ml of Zebrafish Embryo Medium (ZEM). 25 ml of Tricaine working solution were added to each plate of wild-type or mutant zebrafish embryos. The solution was drained and fresh working Tricaine solutions added to each plate every 2 hours starting at 18hpf until 32hpf when embryos were fixed for immunostaining. All stages of the Tricaine drug treatment were performed under low light conditions.

### Galanthamine drug treatment

The stimulation of constant contractions in wild-type fish was performed by using Galanthamine or Nivalin (Enzo Life Sciences, ALX-550-336-M050). Galanthamine stock was prepared by adding 14.4 mg galanthamine to 50 ml of zebrafish embryo media to a final working concentration of 1 mM. 25 ml of working galanthamine solution were added to each experimental plate of wild-type zebrafish embryos at 28hpf. Control wild-type embryo plates had fresh ZEM added to their plates at 28hpf. Control and galanthamine treated embryos were allowed to grow at 28.5°C until 48hpf when they were fixed for immunostaining or flash frozen for RNA extraction.

*Raw data can be found at* *10*.*6084/m9*.*figshare*.*9913700*

## Results

### Myomesin is one of the last structural components organized into the sarcomere of both heart and skeletal muscle

**Fig 2:**
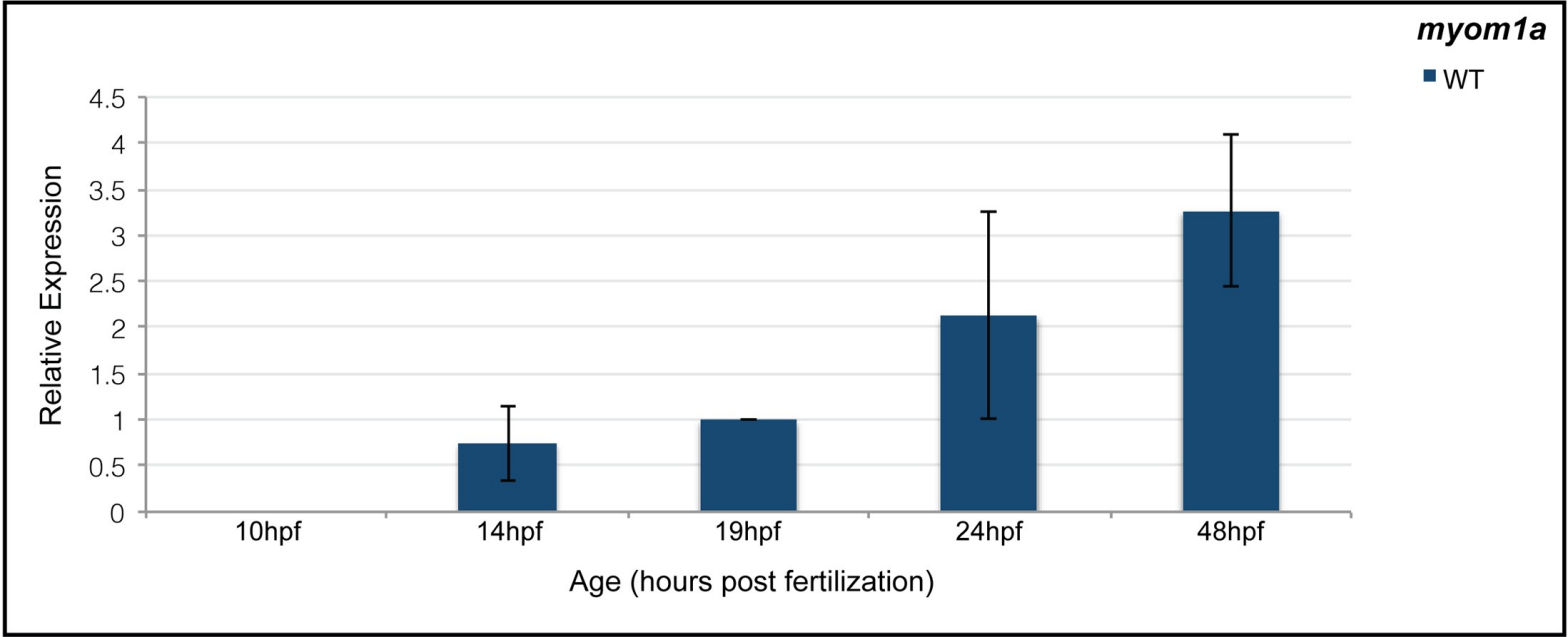
*Myomesin 1a* is expressed at specific stages of myogenesis. qPCR analysis of *myom1a* expression at 10, 14, 19, 24 and 48 hpf in wild-type embryos demonstrated that *myom1a* is not expressed until some time between 10–14 hpf (slow muscle development) and increases throughout myogenesis.

**Fig 3:**
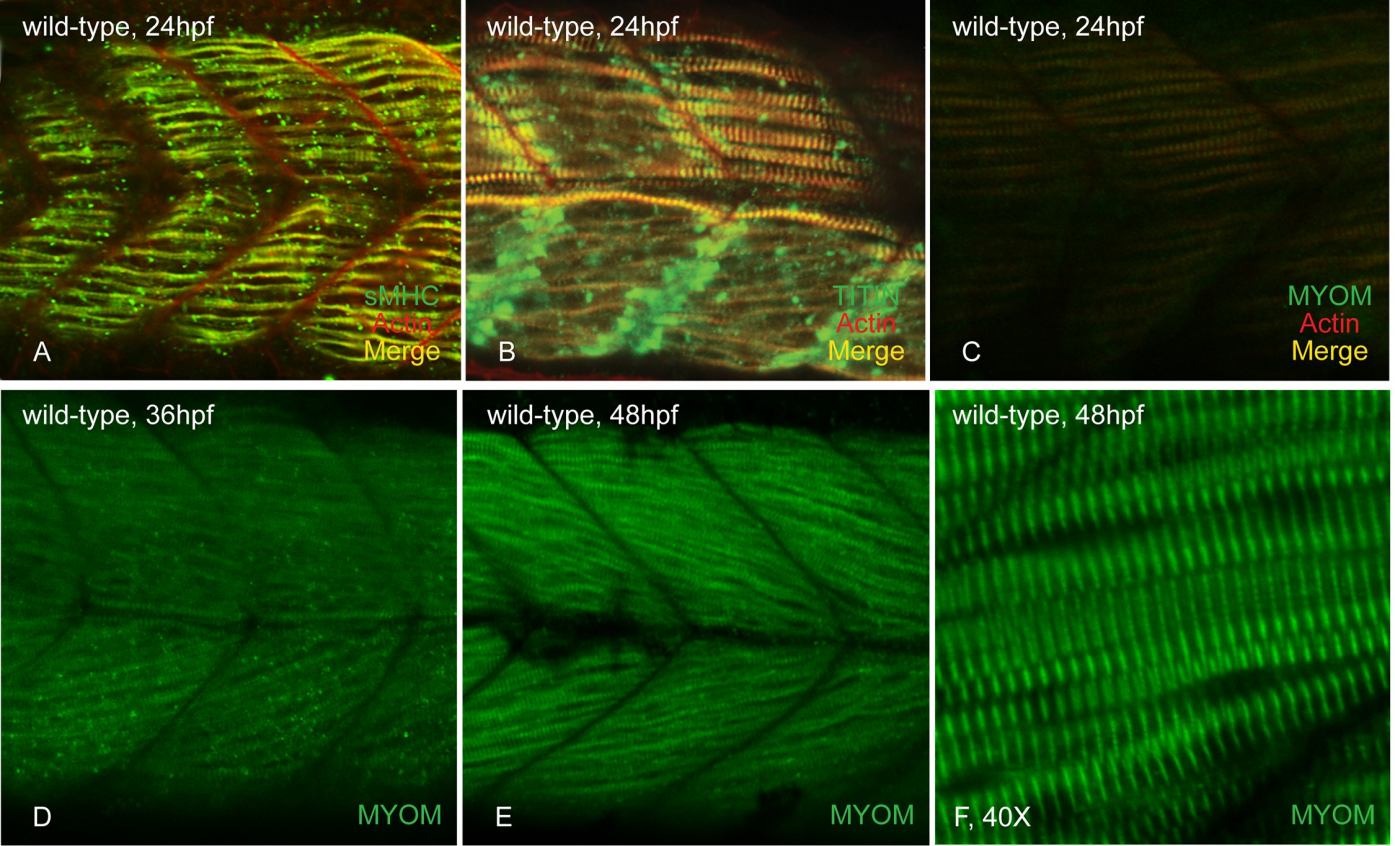
Myomesin is incorporated into skeletal muscle around 24 hpf. At 24 hpf, slow myosin (A) and titin (B) are incorporated and easily visible in the slow muscle fibers of wild-type embryos. Myomesin striations are observed in the parallel slow fibers of caudal somites (C). At 36 hpf, myomesin staining is seen in the developing fast fibers (D) and these striations become more organized and sharp as myogenesis continues at 48 hpf (E&F).

Somitogenesis in zebrafish begins at 10.3 hours post fertilization (hpf) and slow muscle fibers start to differentiate at 14hpf [30,49–51]. Fast fiber development begins at 19hpf, after slow fibers have begun migration to the somite periphery [50]. By 24hpf, the assembly of the contractile sarcomere is nearly complete in both slow and fast muscle tissue with the M-line as one of the last regions to finish [1–4,52]. Based on immunostaining, myomesin is incorporated into the sarcomere by at least 30hpf, but earlier stages were not examined [40]. To determine if myomesin protein is likely to be incorporated into the sarcomere earlier than 30hpf, we examined the expression of *myom1a* transcript along discrete stages of zebrafish myogenesis. *myom1a* transcript is detected at 14hpf (coincident with slow muscle development), 19hpf (when fast muscle development initiates) and continues to be expressed throughout later stages of muscle development (Fig 2). Since *myom1a* expression is detected by 14hpf, we examined early stages (24hpf) of sarcomere assembly and found detectable myomesin immunostaining in the sarcomeres of slow fibers and barely detectable in fast fibers (Fig 3). However at 36hpf, myomesin incorporation is easily observable in the slanted fast fibers of zebrafish tail muscle. Compared with slow myosin and titin striations at 24hpf, myomesin striations are very narrow and only observable in the first 12 somites, suggesting that in comparison to other sarcomere components myosin, actin and titin, myomesin is incorporated last (Fig 3) [22].

**Fig 4:**
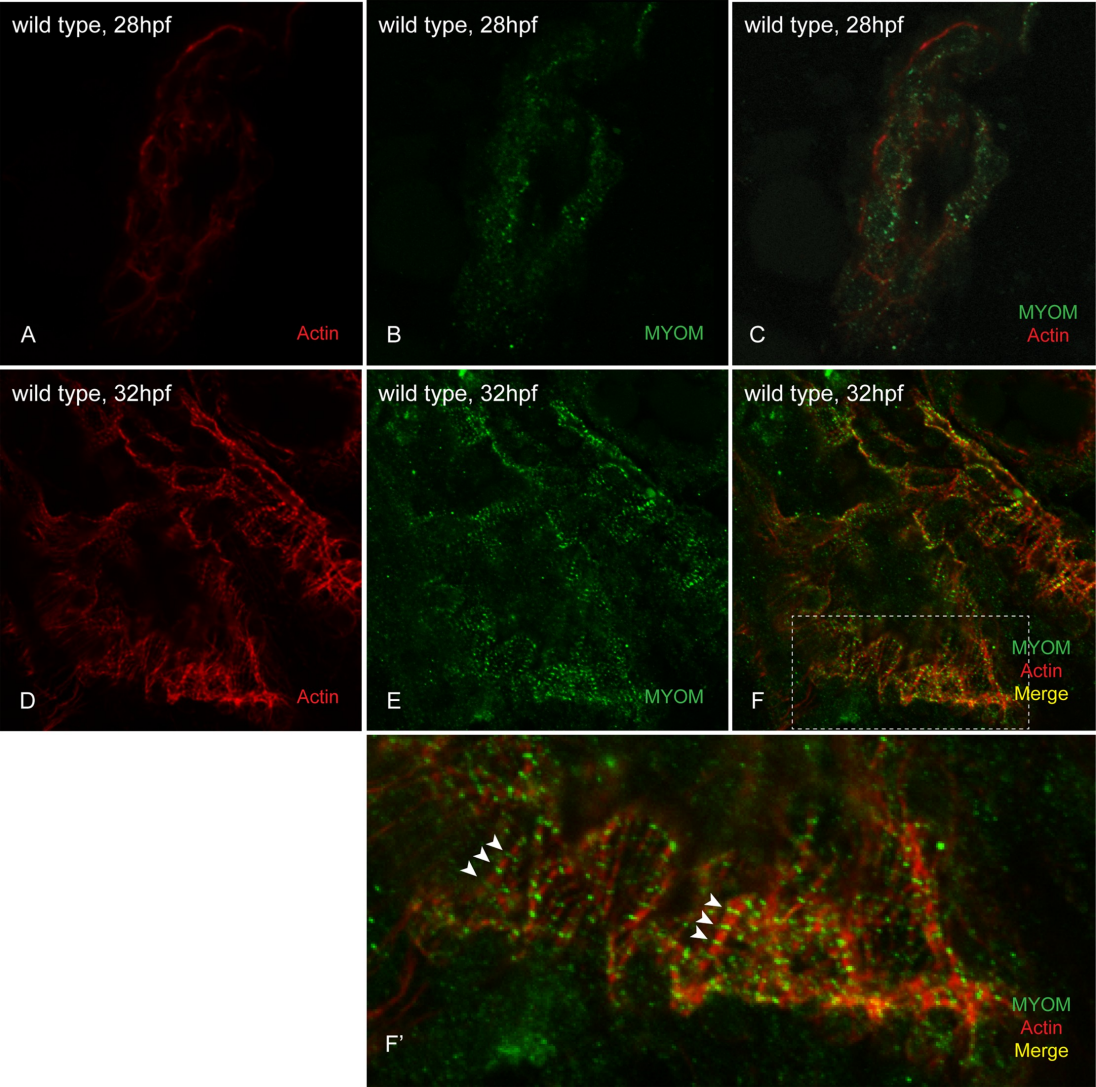
Myomesin is organized into cardiac sarcomeres between 28 and 32hpf. At 28hpf, we could not detect any incorporated myomesin into the sarcomeres of wild-type embryo hearts (A-C). However at 32hpf, myomesin striations are easily visualized in the hearts of wild-type embryos (D-F; white arrowheads in F’).

The zebrafish heart begins beating at ~22hpf and continues through the process of heart looping, where additional cells are added and take on a cardiomyocyte fate [28,53]. We wanted to determine when myomesin incorporates into cardiac sarcomeres to identify a timeline for myomesin-related cardiomyopathies. Due to the fragility and size of the heart, we were not able to analyze hearts younger than 28hpf. However, there was no detectable organization of myomesin into striations within the cardiac sarcomeres at 28hpf (Fig 4). Using immunostaining, we show that myomesin is organized into striations in the hearts of 32hpf wild-type embryos (Fig 4). We hypothesize that as one of the last structures to develop during myofibrillogenesis, the M-band serves as a monitor of sarcomere integrity.

### Myomesin is not present in the M-line of diseased/damaged sarcomeres

**Fig 5:**
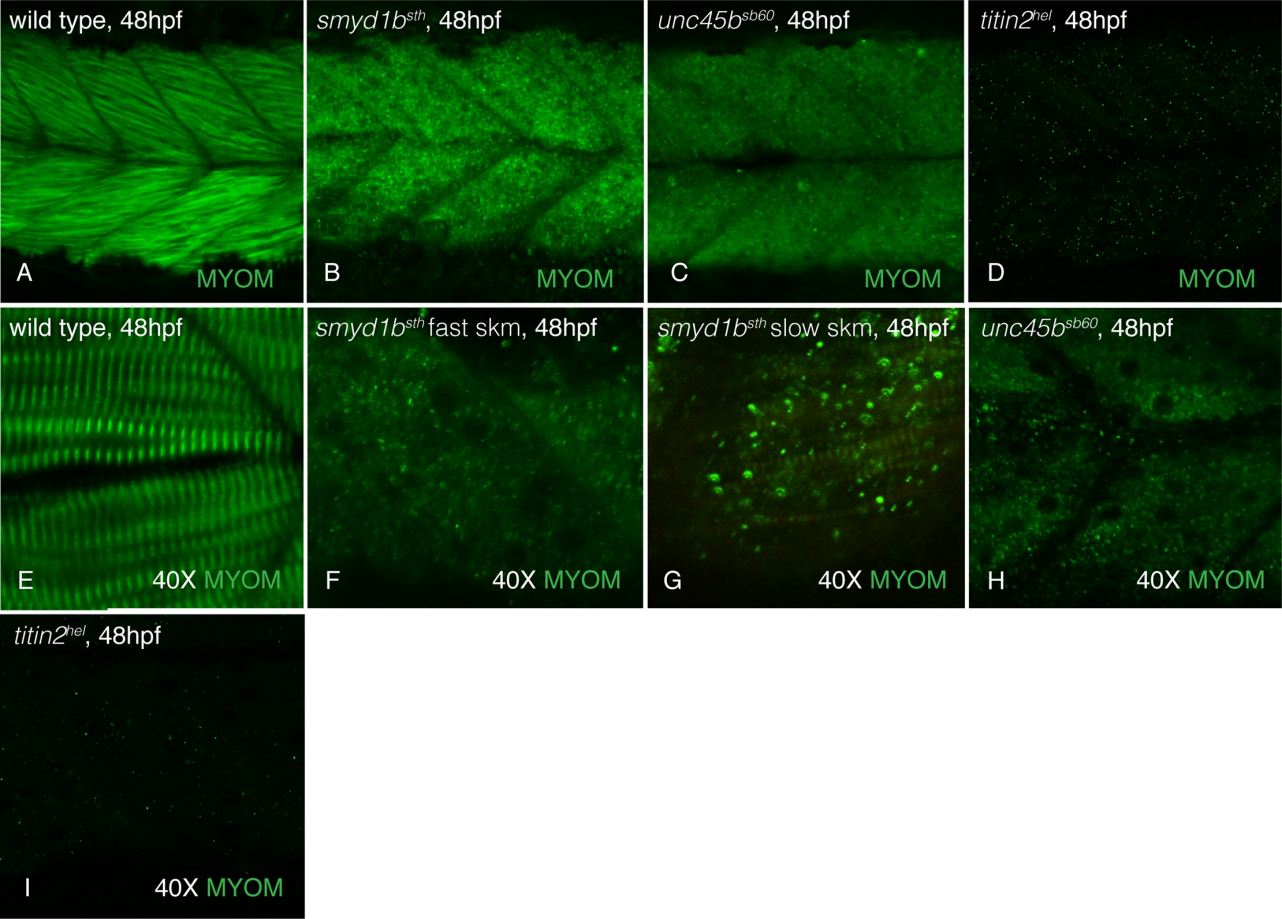
Myomesin is absent in thick filament and titin mutant sarcomeres. At 48hpf, myomesin is incorporated into the slow (perpendicular fibers) and fast (slanted fibers) muscle of wild-type siblings (A&E) while disorganized in *smyd1b*^*tm123a*^ (B&F), *unc45b*^*sb60*^ (C&H) and *titin2*^*tg287*^ (D&I) embryos. Increased magnification (40X) of muscle tissue demonstrates the sharp repeating striations of incorporated myomesin in wild-type siblings (E). *Smyd1b*^*tm123a*^ fast muscle (F; striations are visible in *smyd1b*^*tm123a*^ slow muscle, G), *unc45b*^*sb60*^ (H) and *titin2*^*tg287*^ (I) muscle lack myomesin striations even at 40X magnification.

**Fig 6:**
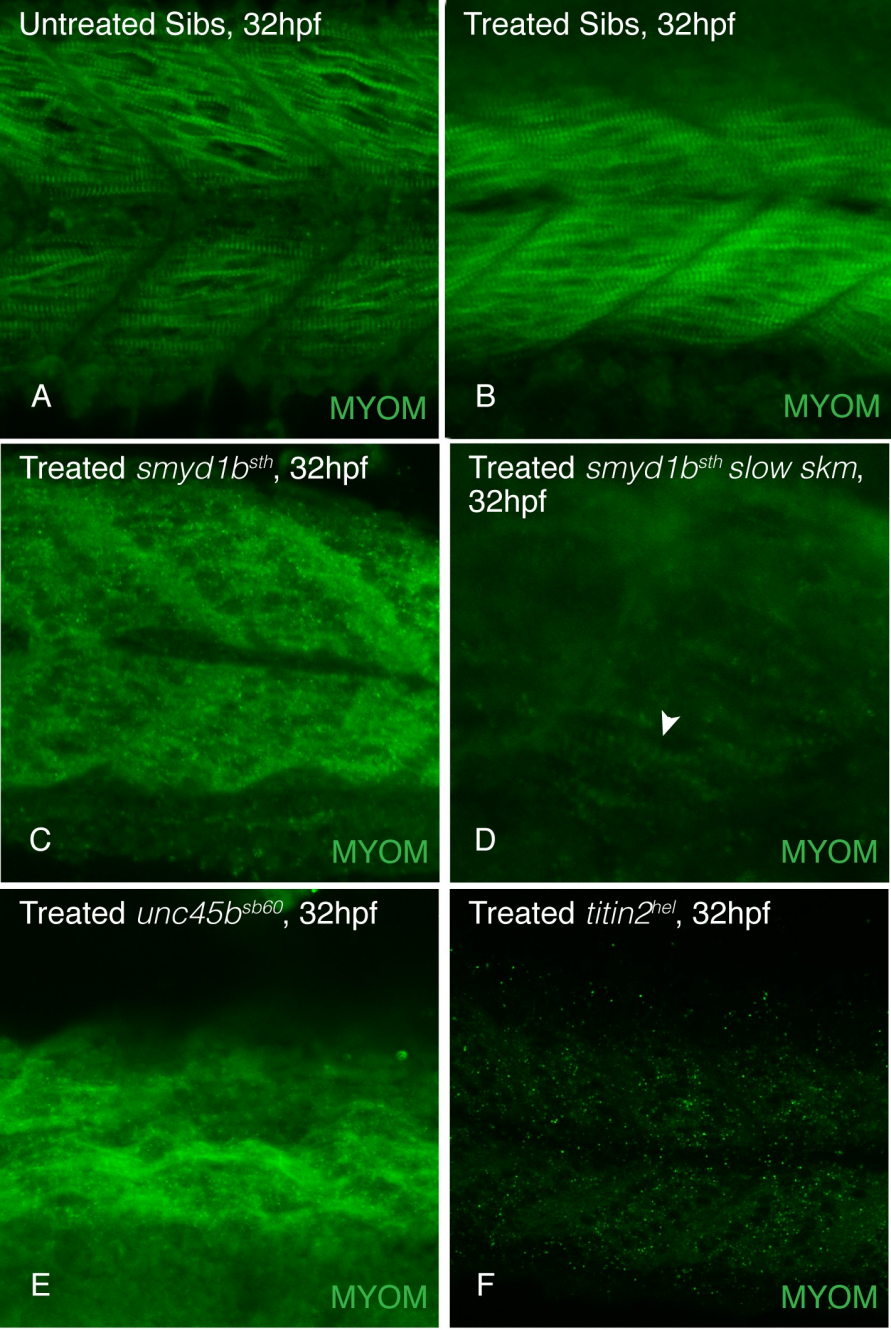
Myosin and titin are required for myomesin incorporation into the sarcomere. Embryos treated with tricaine to inhibit movement and contraction of skeletal sarcomeres from 18 to 32 hpf showed myomesin incorporates normally in untreated (A) and treated (B) wild-type siblings. Myomesin incorporation is absent in tricaine treated *smyd1b*^*tm123a*^ fast muscle (C; myomesin striations are visible in slow muscle (white arrowhead), D) *unc45b*^*sb60*^ (E), and *titin2*^*tg287*^ (F) mutant embryos.

As one test of this hypothesis, we examined the sarcomeres of zebrafish mutant in genes encoding myosin co-chaperones, *unc45b* and *smyd1b*, and a *titin2* mutant, *herzschlag*. *Still heart*, a *smyd1b* mutant, lacks fast myosin incorporation [22] while *steif*, an *unc45b* mutant, lacks both fast and slow myosin incorporation [11,19]. *Herzschlag* mutants, that are predicted to lack the Titin2 C-terminal domain [40], also show a lack of myomesin staining, suggesting the C-terminus of Titin2 is required for myomesin incorporation. These mutants allow us to examine myomesin in conditions where contacts with myosin thick filaments and titin are disrupted. At 48hpf, myomesin is not incorporated into the fast muscle of *still heart* (Fig 5F & 5G) and either fast or slow muscle in *steif* and *herzschlag* mutants (Fig 5). To determine if this loss of myomesin localization in the M-band is due to a loss of connection with myosin and not due to an attempt of the sarcomeres to contract that dislocates myomesin, we anaesthetized wild-type, and the muscle mutant embryos beginning at a time before contractions start (18hpf) to 32hpf and stained for myomesin in the sarcomeres. Anaesthetized *still heart*, *steif* and *herzschlag* mutant embryos did not have myomesin in their M-bands when compared to anaesthetized wild-type embryos (Fig 6), suggesting that it is the lack of myosin in the sarcomere that results in a lack of myomesin incorporation. These results are consistent with a model that without myosin and/or titin connections, myomesin is absent from the sarcomere.

**Fig 7:**
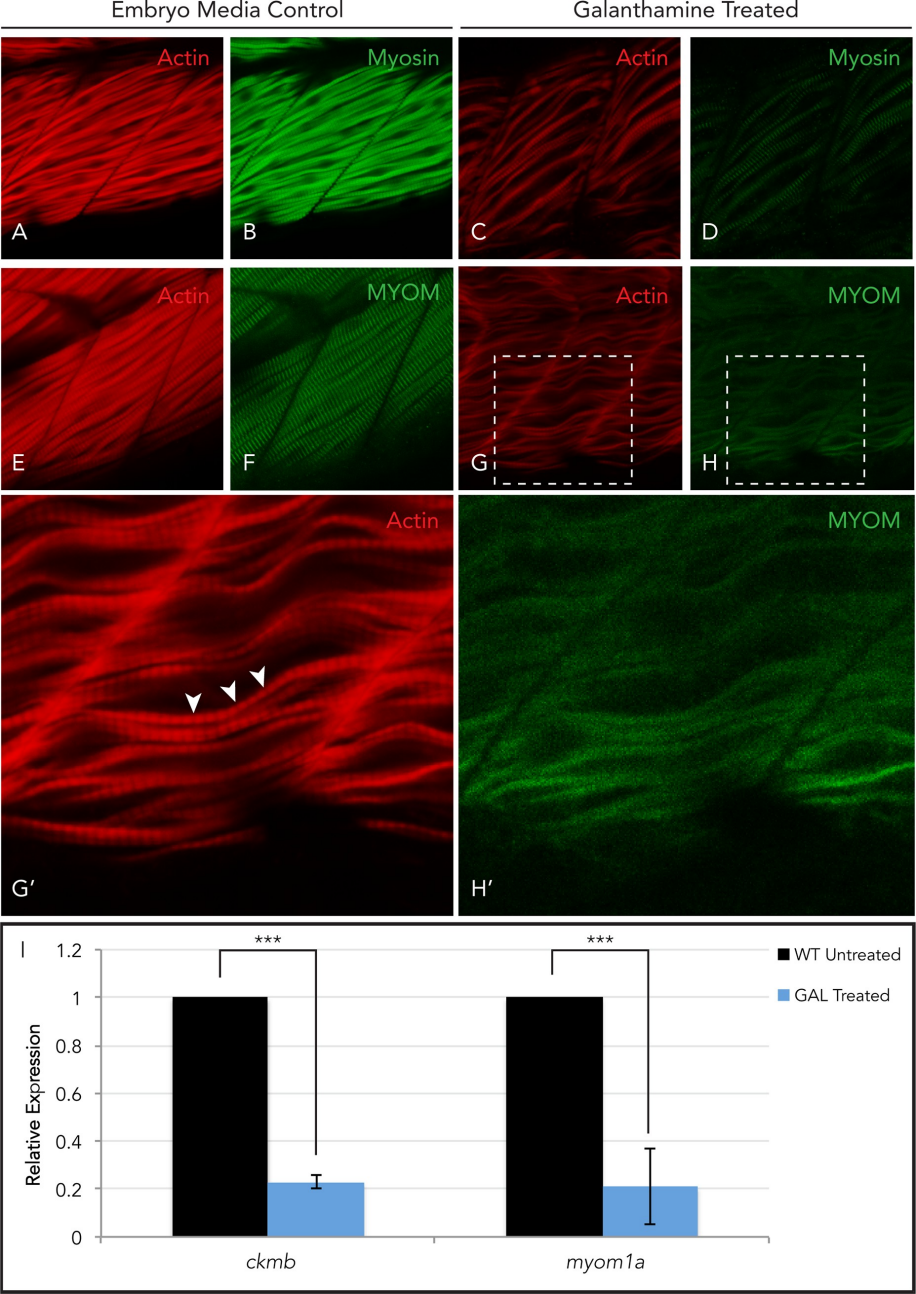
Myomesin is absent in damaged skeletal muscle. Zebrafish embryos treated with galanthamine (GAL) to induce muscle damage by hypercontraction from 28 to 48hpf demonstrated actin and myosin striations in both untreated (A&B) and treated (C&D) wild-type embryos; although GAL treated embryos exhibited fiber disorganization. Myomesin striations are visible in untreated wild-type embryos (E&F) while absent in GAL treated embryo muscle (G&H) even though actin striations, indicative of intact sarcomeres, are present (G’, white arrowheads; H’). qPCR analysis of *ckmb* and *myom1a* expression in galanthamine treated and untreated wild-type embryos demonstrated a significant decrease in the expression of both M-band genes at 48hpf (I). (Error bars are standard deviation. Expression is normalized using *ef1a* and wild-type at 48hpf).

We have shown that myomesin cannot localize to the M-band of diseased sarcomeres but we also wanted to test if myomesin would be absent from damaged muscle that assembled normally. To test this hypothesis, we chemically treated wild-type zebrafish embryos with galanthamine (GAL), a well-known inhibitor of acetylcholinesterase [54,55]. Galanthamine causes hypercontractility in zebrafish skeletal muscle leading to contraction-induced degeneration or dystrophy [54,56]. Wild-type embryos were treated at 28hpf (after skeletal sarcomere assembly) to 48hpf and analyzed for myomesin staining in the sarcomeres. GAL-treated wild-type embryos displayed disorganized myofibers with evident actin and myosin striations but myomesin striations were absent when compared to embryo media control embryos (Fig 7). This suggests that in normally developed muscle that undergoes significant cellular damage, such as in muscular dystrophies, myomesin is absent from the sarcomere and that this occurs even when myosin is still present.

Since we did not observe myomesin in the M-line of GAL-treated embryos, we wanted to determine if the expression of myom1a was affected by drug-induced hyper-contractility. After 20 hours of galanthamine treatment, *myom1a* expression was significantly down-regulated in drug-induced damaged muscle (Fig 7I). When compared to another M-band localized protein, muscle creatine kinase b, which is used as a biomarker for damaged muscle, we observed a significant reduction in *ckmb* expression in GAL-treated embryos at 48hpf (Fig 7I). This would suggest the damage caused by hypercontraction activates a transcriptional feedback that reduces the expression and organization of M-band components. These transcriptional changes may provide insight into the mechanism behind hypertrophic cardiomyopathy disease progression.

### Myomesin responds to defects in sarcomere assembly before other markers of muscle damage

**Fig 8:**
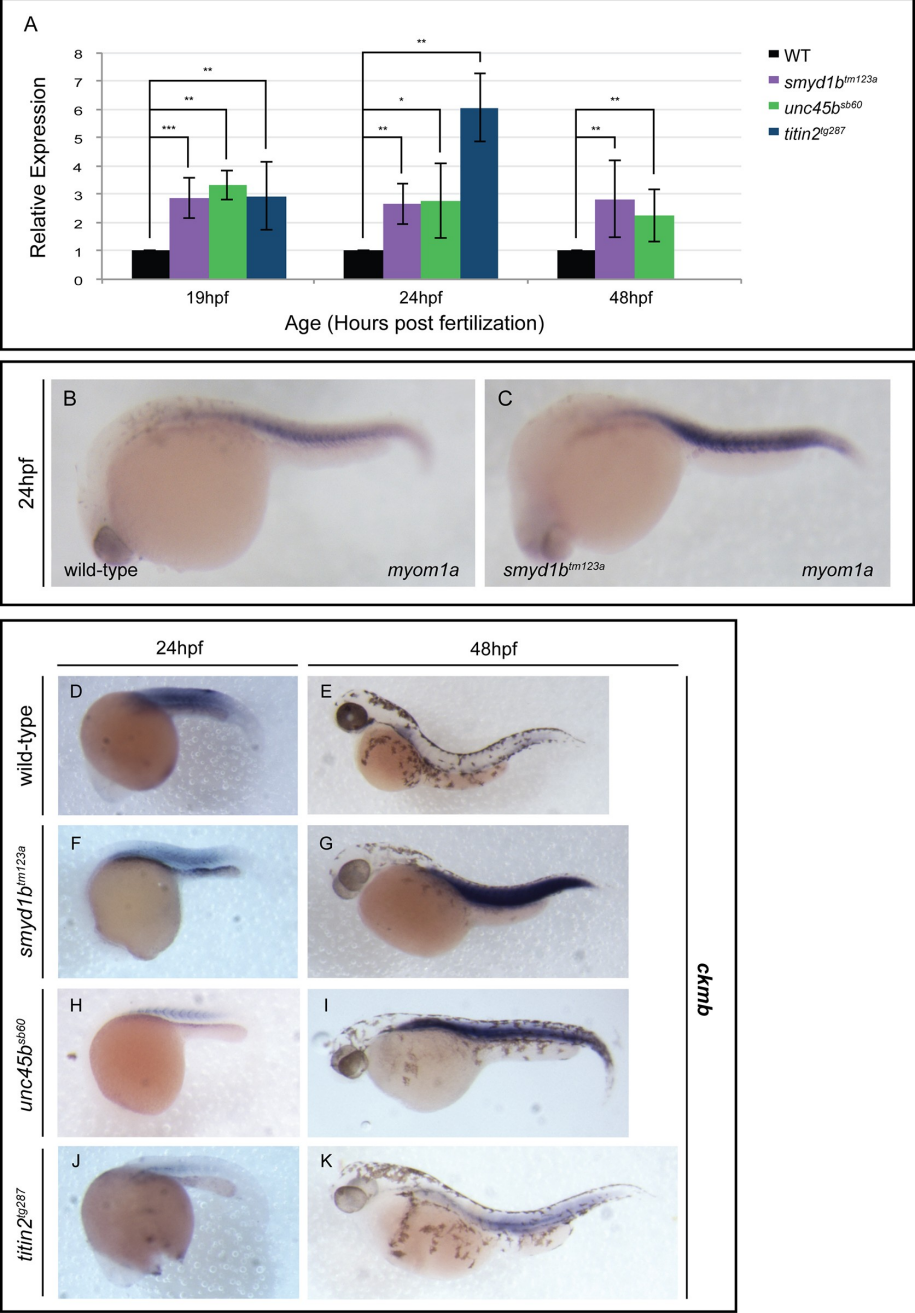
*Myomesin1a* is significantly upregulated at early stages of myogenesis in muscle mutants. qPCR analysis at 19, 24 and 48 hpf revealed a statistically significant up-regulation of *myom1a* expression in *smyd1b*^*tm123a*^, *unc45b*^*sb60*^ and *titin2*^*tg287*^ mutants relative to wild-type embryos at identical stages (A). At 24 hpf, *in situ* hybridization demonstrated somite-restricted *myom1a* expression in wild-type siblings (B), which is increased in *smyd1b*^*tm123a*^ embryos (C). At 24 hpf, wild-type (D) and *smyd1b*^*tm123a*^ (E) embryos display similar levels of muscle *creatine kinase b* (*ckmb*) expression in their skeletal muscle. Compared to wild-type embryos at 24 hpf (D), *unc45b*^*sb60*^ (H) and *titin2*^*tg287*^ (J) show a significant decrease in *ckmb* expression. At 48 hpf, *smyd1b*^*tm123a*^ (G), *unc45b*^*sb60*^ (I) and *titin2*^*tg287*^ (K) embryos demonstrated increased *ckmb* expression in their skeletal muscle when compared to wild-type siblings (E). (Error bars are standard deviation. Expression is normalized using *ef1a* and wild-type at corresponding stages).

If the M band acts as a monitor of sarcomere integrity, we hypothesize that sarcomeres that cannot incorporate, or dislodge myomesin, will display a transcriptional response similar to that of other muscle damage response pathways. The chaperones *hsp90a1*, *unc45b* and *smyd1b* are transcriptionally up-regulated in response to misfolded myosins at 24 and 48hpf in *still heart* and *steif* mutants [11,19,22]. This response seems to be due to a threshold level of misfolded myosins that cause Hsp90a1-bound Hsf1 to translocate to the nucleus to induce a chaperone response [57]. Our model predicts that if myomesin cannot make appropriate interactions with proteins in the M-band, it fails to incorporate, and we would see a positive feedback on *myom1a* transcription to help repair the defective sarcomere assembly. We show that the expression of *myom1a* is significantly up-regulated at 19hpf and older stages in still heart, steif and herzschlag mutants (Fig 8A–8C), an increase that is not observed in chaperone expression until 24hpf [22].

One of the most well-known and used biomarkers for sarcomere damage is muscle creatine kinase [58]. Unlike myomesin 3, creatine kinase serum levels increase from normal exercise or diseased muscle while myom3 is specific to sarcomere damage [45]. Since *myom1a* shows increased expression early in congenital myopathies, we wanted to test whether *myom1a* is also a specific biomarker for sarcomere damage when compared to muscle creatine kinase in zebrafish at a tissue level. When compared to *myom1a*, which shows increased expression starting at 19hpf and onwards, *muscle creatine kinase b* showed increased expression at 48hpf but not at 24hpf (Fig 8D–8K), suggesting muscle creatine kinase is either less specific to muscle disease than Myomesin 1a or that muscle creatine kinase expression is regulated by a separate pathway activated in later stages of sarcomere damage. This supports the model proposed by Rouillon and colleagues [45] that myomesin assays could detect muscle disease earlier than current markers, but expands this assay to myopathies beyond muscular dystrophies. However, our expression analysis of *myom1a* and *ckmb* in hypercontractile muscle displayed an opposite trend for both genes, which had decreased expression after 20 hours of galanthamine treatment (Fig 7). This corroborates the changes in myomesin expression observed in rats with hypertrophic cardiomyopathy with increased activity of their cardiac sarcomeres [59].

## Discussion

We hypothesized that the M line acts as a monitor of sarcomere integrity. In support of our hypothesis, we show that myomesin is one of the last proteins to incorporate into the developing sarcomere and that this incorporation requires connections with myosin and Titin2. In diseased sarcomeres, myomesin is missing and a positive feedback myomesin transcriptional response is observed as early as 19hpf. As this response occurs earlier than known markers of muscle damage, chaperones and the clinically used, creatine kinase, we and others support myomesin assays as a early and specific marker of muscle damage [26,36,45,59–62].

Myomesin makes physical contact with myosin heavy chains, titin and obscurin and is required to distribute contractile forces across thick filaments [35–37,39,42]. We focused on the effect that an absence of myosin or the C-terminus of titin has on myomesin. It could be that the loss of one or both of these protein interactions allows myomesin to translocate to the cytoplasm or nucleus [63,64]. Support for this displacement of myomesin comes from the lack of detectable myomesin incorporated into diseased/damaged sarcomeres (Figs 5 & 7) and the high concentration of myomesin 3 protein in the sera of Duchenne muscular dystrophy, Limb-girdle muscular dystrophy and dilated cardiomyopathy patients and animal models [45,60–62,65]. We showed that the absence of myosin or the C-terminus of titin can prevent myomesin from incorporating into the sarcomere and that it is not paralysis or contractions in a defective sarcomere that displace myomesin in these fish (Fig 6). In addition to the absence of myomesin in improperly developed sarcomeres, we demonstrated that myomesin is absent from the sarcomere when muscle undergoes significant cellular damage (Fig 7). Despite our galanthamine treatment being shorter than the typical 3 day treatment, the effects of GAL-induced hypercontraction were evident in the treated embryos, suggesting myomesin translocates from the sarcomere in the early stages of muscle wasting and degeneration. While this study focused on myom1a at the tissue level, future work should focus on assaying the sera of embryonic higher vertebrate models for myomesin 1(a) in drug-treated or dystrophic animals.

*Myomesin1a* mRNA expression is increased much earlier than the myosin chaperones in the *smyd1b*^*tm123a*^ mutant, defective in thick filament assembly (Fig 8). *Myom1a* expression is also increased in *titin2*^*tg287*^ mutants before sarcomere damage is detectable. This early up-regulation in *myom1a* gene transcription could suggest that myomesin1a is part of a sarcomere integrity/repair pathway. Several other integrity monitors exist in the M-band such as myomasp/LRCC39 and Murf2, which isolate Serum Response Factor (SRF) to the sarcomere in healthy muscle [66,67]. Myomasp physically interacts with myosin, myomesin and SRF while Murf2 binds SRF and titin, both regulating SRF-mediated transcriptionduring normal sarcomere assembly and function [67,68]. If myosin is damaged, or absent, myomasp cooperates withSRF to activate SRF target genes such as myosin heavy chains, myomesin and smyd1b, suggesting the activation of these genes is to aid in rebuilding or repairing the sarcomere [68–71]. If titin is absent, damaged or inactive, MuRF2 translocates to the nucleus to degrade nuclear-localized SRF, which then supresses the SRF transcriptional pathway. The SRF-mediated response pathway could explain the early increase of *myom1a* gene transcription that we observed in myosin and titin mutants where myompasp may activate the SRF signalling pathway in conjunction with the activation of the Misfolded Myosin Response [19,22,40,57,68]. If the increased *myomesin1a* expression were secondary to the SRF-mediated response pathway, it would be essential to test the SRF response in a *myom1a* mutant where, hypothetically, myosin and titin should remain intact at the M-line but Myomasp would be incapable of making all of the necessary protein connections. This would provide an explanation for the results seen in human Dilated Cardiomyopathy (DCM) patients, which have defects in sarcomere structure or force sensing mechanisms in their cardiac muscle, who display significantly increased expression of the EH-*myom1a* transcript and immunostaining of EH-MYOM1a in their heart tissue [36,60].

Further analysis is required to complete our understanding of the signaling pathways involved with myomesin and muscular dystrophies. The data presented here supports the lack of myomesin in dystrophic muscle (Fig 5)[62] with *myom1a* transcription significantly upregulated (Fig 8), suggesting post-transcriptional regulation of myomesin. Our titin2 mutant lacks a C-terminus after 19hpf, which means MuRF2 and associated factors cannot localize to the kinase domain of titin2 [67]. Whether this affects the SRF-mediated signaling pathway or other transcriptional regulators of sarcomere components is unknown.

Taken together, myomesin1a is a promising early detection marker for diseased or damaged sarcomeres and is part of a specific early detection pathway (Fig 8D–8G). Recent research has started to look at serum and urine levels of titin as a biomarker for muscle disease, supporting the movement of early and specific disease detection to structural components of the sarcomere [72–75]. This work in combination with research of myomesin paralogs, supports the use of myomesin assays as early detection methods of muscle damage in myopathy patients as well as a measure of the effectiveness of treatments in muscle disease.
